# Individual placement and support and employment in personality disorders: a registry based cohort study

**DOI:** 10.1186/s12888-022-03823-4

**Published:** 2022-03-17

**Authors:** T. T. Juurlink, F. Lamers, H. J. F. van Marle, W. Zwinkels, M. A. Spijkerman, A. T. F. Beekman, J. R. Anema

**Affiliations:** 1 Amsterdam UMC, Vrije Universiteit, Social Medicine, Amsterdam Public Health Research Institute, De Boelelaan, 1117 Amsterdam, The Netherlands; 2 Amsterdam UMC, Vrije Universiteit, Psychiatry, Amsterdam Public Health Research Institute, Amsterdam, The Netherlands; 3 GGZ in Geest Specialized Mental Health Care, Amsterdam, The Netherlands; 4 Amsterdam UMC, Vrije Universiteit, Psychiatry, Amsterdam Neuroscience, Amsterdam, The Netherlands; 5 Epsilon Research, Leiden, The Netherlands; 6 UWV, Dutch Social Security Administration, Amsterdam, The Netherlands

**Keywords:** Employment, Individual placement and support, Netherlands, Personality disorder, Registry based cohort

## Abstract

**Background:**

To explore the relative impact of Individual Placement and Support (IPS) in patients with personality disorders (PDs) as compared to patients with other mental disorders.

**Methods:**

Data from the Dutch Employee Insurance Agency of participants enrolled in a national IPS trajectory between 2008 and 2018 were linked to corresponding data on employment outcomes, diagnostic and sociodemographic information from Statistics Netherlands. This resulted in a sample of 335 participants with PDs who could be compared with 1073 participants with other mental disorders.

**Results:**

Participants with PD just as often found competitive employment as participants with other mental disorders (37.6% vs. 38.0%, OR_adjusted_ = 0.97, 95% confidence interval (CI) 0.74 to 1.27). The median time to gaining employment for those gaining employment (37.9%) was 195.5 days (mean number of days 252.5) in the PD group and 178.5 days (mean number of days 234.6) in the other mental disorders group (HR_adjusted_ = 0.95, 95% CI 0.77 to 1.18). Also, total number of hours paid for competitive employment did not differ significantly between groups (median hours 686.5 vs 781.5, IRR_adjusted_ = 0.85 95% CI 0.69 to 1.05).

**Conclusions:**

Based on this study, which includes the largest sample of patients with PDs in any published IPS study, IPS seems to result in an equal percentage of patients with PDs and other mental disorders, gaining and maintaining employment. Although future studies should determine whether PD-specific adaptations to IPS are useful, our findings indicate that IPS could be an effective way to increase employment outcomes in PDs. This is important because the enormous societal costs of PDs are largely driven by loss of economic productivity, and because clinical recovery in PDs is suggested to be enhanced when patients are employed.

**Supplementary Information:**

The online version contains supplementary material available at 10.1186/s12888-022-03823-4.

## Background

Personality disorders (PDs) are severe mental illnesses characterized by deviating patterns of inner experience and behaviour in the areas of cognition, affect regulation, interpersonal- and self-functioning, and impulse control. Typically, maladaptive behavioural patterns are inflexible, present across a broad range of social and personal situations and cause considerable personal distress [1]. PDs are associated with impaired occupational functioning and unemployment [2–4], and although symptoms of PDs tend to diminish over time and treatment of PDs is effective, occupational functioning tends to remain poor irrespective of symptom remission [5–7]. Still, few studies report on the factors that contribute to occupational dysfunction in PDs. In our previous qualitative study exploring barriers and facilitators to employment in borderline personality disorder (BPD), we show that maintaining employment is considered more difficult than gaining employment by both patients and professionals [8]. In this study, the characteristics of BPD that impeded occupational functioning mainly related to interpersonal functioning and emotion regulation. Considering all PDs, the shared hallmark symptom of having difficulty with interpersonal relationships is suggested to be the central factor of occupational dysfunction [9]. This is different in for example, psychotic disorders, where next to interpersonal and cognitive functioning problems [10, 11] positive and negative symptoms and low expectations hindered employment [12], or in affective disorders, where a lack of motivation and proactiveness due to depressive symptoms might prevent successful employment [13]. However, perspectives on barriers to employment can differ between patients and clinicians [14].

Individual Placement and Support (IPS) is a well-established evidence-based method of supported employment based on the first *place, then train* principle, originally developed to support patients with severe mental illnesses [15]. The method focusses on participants’ preferences and the assumption that everyone willing to gain employment can find regular paid employment [16–18]. To receive IPS in the Netherlands, participants need to be in mental health treatment, unemployed, and express a wish to gain regular paid employment. Ample evidence shows effectiveness of IPS in various groups, such as patients with psychotic and affective disorders, patients within forensic mental health care, patients with substance use, musculoskeletal and neurological disorders, and veterans [17, 19, 20]. Also, recent studies show that IPS is effective in young people with moderate mental illnesses, and living within European policy contexts characterized by high job security and a comprehensive welfare system comparable to the context of the present study [21, 22]. IPS, however, has not been directly studied in patients with PDs. In an exploratory secondary analysis of a small randomized controlled trial of IPS in a large mixed patient group in the Netherlands, no difference in effectiveness of IPS was found between patients with PDs and patients with other mental disorders [23]. This suggests that IPS may be effective also for PDs, although the total number of PD patients in this study was too low to draw any definitive conclusions.

In this study we link datasets from the Employee Insurance Agency (UWV), holding data of all participants enrolled in a national IPS trajectory in the Netherlands and Statistics Netherlands (CBS) which holds register data on employment outcomes, diagnostic and sociodemographic information of the corresponding participants. This provides a unique opportunity to explore the relative impact of IPS in patients with PDs as compared with patients with other disorders in a large cohort of well-documented cases. Specifically, we test whether both groups of patients differ on: i) gaining employment for at least one hour during study follow-up, ii) time in days to gaining employment, and iii) duration of employment in cumulative number of hours worked (since maintenance of employment is a key concern in patients with PDs).

## Methods

### Design

A registry-based cohort study examining employment outcomes in records of IPS participants, comparing effectiveness between participants with a PD and participants with other mental disorders. Data from the Employee Insurance Agency (UWV) containing information on enrolment and commencement of a national IPS trajectory with inclusion from 2008 to 2018 were linked to data of the Netherlands and Statistics Netherlands (CBS) containing employment records from 2008 to mid-2019, and records of DSM-IV diagnosis from 2011 to 2016.

### Participants and data linkage

**Fig. 1:**
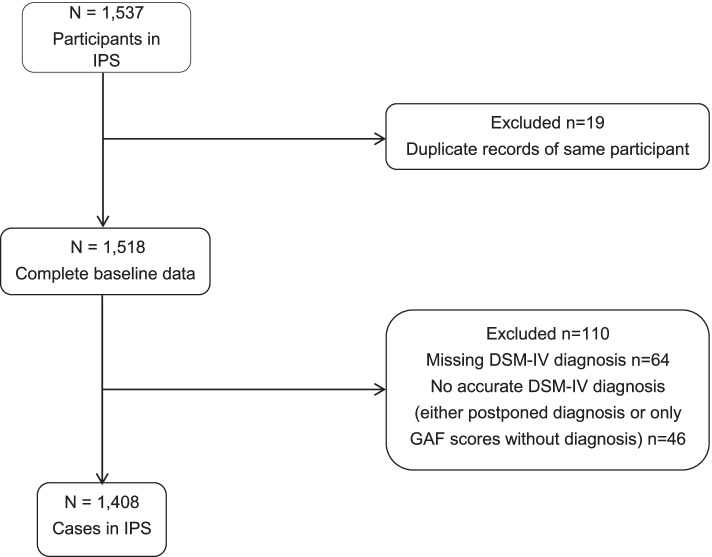
Flowchart of excluded cases and study sample

The current study population was restricted to IPS participants in the UWV registry of whom a DSM-IV diagnosis could be retrieved from the CBS dataset (see Fig. 1). Using anonymized personal identification numbers, data of the CBS were linked to the anonymized IPS records of the UWV from 2008 to 2018. All participants in IPS with a DSM diagnosis of any mental disorder classified as a severe mental illness including among others: psychotic, depressive, affective, and pervasive developmental disorders were included in the analyses and defined as other mental disorder group. Data on employment records of the CBS were from January 2008 until June 2019 and included: 1) dates of entrance into employment, 2) type of employment (competitive or sheltered employment), 3) number of hours worked in paid employment including overtime, without surcharges and time off for overtime hours. Diagnostic information was based on mental health care registered DSM-IV data from the CBS from 2011 to 2016. The diagnosis are based on chart-diagnoses, mostly based on clinical judgement by clinicians. However, all diagnoses are based on DSM-IV criteria. In the Netherlands, a DSM-IV diagnosis can only be given by a certified clinician (psychiatrist or psychologist). For the IPS trajectories starting between 2008 and 2011 the first available DSM-IV diagnosis following the start of IPS was used. Conversely, for the IPS trajectories starting after 2016, the last available diagnosis was used. PD diagnosis was a dichotomous variable, counting any occurrence of a main or secondary PD diagnoses recorded between 2011 and 2016. The group never receiving any PD diagnosis was assigned to the other severe mental illness (SMI) group. We opted to assign all participants who were registered to have a PD diagnosis at any time during the registration period to the PD group because personality psychopathology in adults tends to be more stable as compared to symptoms of axis-I psychopathology [24], and misclassification of PDs is common [25]. A few participants entered an IPS trajectory twice, which could potentially affect the outcomes on employment because these individuals might have gained experience in gaining and maintaining employment from their first IPS trajectory. Therefore, the outcomes of the second trajectories were excluded from the analyses (*n* = 25). In the Netherlands, and according to IPS model fidelity, IPS participants are supported for three years, which subsequently was the follow-up period for the present study.

### Measures

The primary outcome was the proportion of participants who were competitively employed during the study follow-up. This was dichotomously measured as having worked in competitive employment for one hour or more. Competitive employment was defined as having a paid job (not a sheltered job) based on CBS database records of paid contract hours. Secondary outcome measures were time to gaining employment and duration of employment. Time to gaining employment was based on the number of days between starting the IPS trajectory and starting competitive employment as identified from the first payment record. Duration of employment was based on the cumulative number of hours paid in competitive employment among those in competitive employment during IPS follow-up.

### Covariates

Like previous studies on occupational functioning in mental disorders, the following potentially confounding variables were included: gender, age, nationality (Dutch, Western, non-Western) and employment history (competitively employed in the past 5 years before entering the IPS trajectory yes/no) [26–29]. Although education level is associated with occupational functioning in mental disorders [30], unfortunately we did not have access to this information and could not analyse education as a putative confounding variable in the present study.

### Statistical analysis

Sample characteristics were explored and presented as frequencies, and percentages, with medians and interquartile ranges (for non-normal distributed variables). Differences between groups (PD versus other mental disorders) were tested with Chi-square or Mann Whitney U tests. Additionally, we described and tested differences in gaining employment between different patient groups (schizophrenic and psychotic disorders, personality disorders and other mental disorders). The primary outcome measure - gaining competitive employment for at least one hour during the IPS trajectory - was analysed with logistic regression. Time to employment in number of days was studied with Cox proportional hazards models of which the assumptions were checked and satisfied. The total sample was taken into account, participants were right-censored if they did not gain a job within the three-year follow-up. Also, participants that were lost to follow-up were censored. Within those in competitive employment (*n* = 534), the cumulative number of hours worked was compared between groups with Poisson regression by means of a negative binomial distribution due to underdispersion. For this analysis the values of the outcome variable need to be round integers. Therefore, the 13% with decimal values were rounded to the nearest integer. Furthermore, the negative binomial regression was corrected for the number of weeks in follow-up because the amount of time in follow-up might bias the outcome. The pattern of follow-up did not significantly differ between groups. Finally, all analyses were run both unadjusted and adjusted for gender, age, nationality and employment history.

A few factors could potentially influence analyses. First, for a number of participants the IPS trajectory follow-up was still ongoing. These cases might gain employment in the future and therefore the long-term effects of IPS in the present study may be underestimated. Second, as previously described the group ever reporting a PD was assigned to the PD group. This group, however, might have reported another mental disorder before, during or after the IPS trajectory which might bias the accuracy of the results. Therefore, sensitivity analyses were conducted to evaluate the robustness of our findings. For this, all analyses described above were also conducted: 1) with exclusion of cases of which the IPS trajectory was still ongoing (*n* = 66 in PD, *n* = 194 in other SMI, total *n* = 1148), and 2) with exclusion of cases of which the last registered diagnostic information was not a PD although they were assigned to the PD group (96 cases, 28.7% of total sample) (*n* = 1312). In addition, we once ran the analyses including 25 cases (*n* = 1 PD, *n* = 24 SMI) who had 2 IPS trajectories. All statistical analyses were performed using SPSS version 24.0 or Rstudio version 3.6.2.

## Results

### Characteristics of the study population

The study population included 1408 participants with a IPS trajectory that was initiated between 2008 and 2018 in the Netherlands. Of these, 335 participants had a PD diagnosis and 1073 participants had another mental disorder as diagnosis. Table 1 presents the characteristics of both groups. The largest proportion of participants in both groups was between 26 to 35 years of age and had the Dutch nationality. Gender, age, and nationality differed significantly between groups.

**Table 1:** Sample characteristics of IPS participants (*n* = 1408)

	PD	Other SMI	*p-*value
	*N* = 335	*N* = 1073	
Female gender, n (%)	192 (57.3)	309 (28.8)	**<0.01**
Age, mean (SD)	37.17 (8.8)	35.3 (9.0)	**<0.01**
19–25 years, n (%),	19 (5.7)	155 (14.4)	
26–35 years, n (%),	143 (42.7)	442 (41.2)	
36–45 years, n (%),	106 (31.6)	310 (28.9)	
46–64 years, n (%),	67 (20.0)	166 (15.5)	
Nationality, n (%)			**<0.01**
Dutch	277 (82.7)	714 (66.5)	
Western immigrant	29 (8.7)	117 (10.9)	
Non-Western immigrant	29 (8.7)	242 (22.6)	
Employment history (employed in past 5 years), n (%)	184 (54.9)	574 (53.5)	0.65

*PD* Personality disorders

*Other SMI* other severe mental illness

Significant *p*-values highlighted in bold

Additionally, three diagnostic groups were compared to examine potential differences in finding competitive employment in IPS (Table 2). The largest number of IPS participants was diagnosed with schizophrenia or other psychotic disorders. Additional file 5 shows the frequency of the different personality disorder diagnoses per cluster (A, B, C) and PD not otherwise specified.

**Table 2:** Descriptive characteristics of gaining employment per diagnosis group (*n* = 1408)

DSM-IV diagnosis	Total n employed (%)	Female gender n (%)	Male gender n (%)	*p*-value
				0.30
Schizophrenia and psychotic disorders	237 (36.2)	60 (30.3)	177 (52.7)	
Personality disorders	126 (37.6)	75 (37.9)	51 (15.2)	
Other mental disorders^a^	171 (40.9)	63 (31.8)	108 (32.1)	

^a^Among others affective and pervasive developmental disorders

### Engagement in employment

At any time during the IPS follow-up, 37.6% of the PD participants were competitively employed for at least one hour versus 38.0% of those with other mental disorders. This is a negligible difference, resulting in a non-significant odds ratio (OR = 0.98, 95% CI 0.76 to 1.27, *p* = 0.89) (Table 3). Although age (OR = 0.98, 95% CI 0.97 to 0.99, *p* = <0.01) and employment history (OR = 2.15, 95% CI 1.72 to 2.69, *p* = <0.01) were significantly associated with the effect of IPS, adjustment for age, gender, nationality and employment history did not alter the results comparing between groups (OR = 0.97, 95% CI 0.74 to 1.27, *p* = 0.83).

**Table 3:** Employment outcomes of IPS participants and associations of employment with group (*n* = 1408)

	PD			Other SMI		
Finding competitive employment, n (%)	126 (37.6)			408 (38.0)		
	Model 1^a^			Model 2^a^		
	OR	95% CI	*p*-value	OR	95% CI	*p*-value
PD	0.98	0.76–1.27	0.89	0.97	0.74–1.27	0.83
Age	n/a	n/a	n/a	0.98	0.97–0.99	**<0.01**
Female gender	n/a	n/a	n/a	0.88	0.69–1.11	0.28
Dutch nationality	n/a	n/a	n/a	1.00	0.87–1.16	0.96
Employment history	n/a	n/a	n/a	2.15	1.72–2.69	**<0.01**
Time to gaining competitive employment in days, median (IQR) worker sample (*n* = 534)	195,5 (74.0–379.0)			178,5 (76.0–341.0)		
Time to gaining competitive employment in days, mean (SD) worker sample (*n* = 534)	252.5 (223.7)			234.6 (204.1)		
Time to gaining competitive employment in days, total sample	Model 1^b^			Model 2^b^		
	HR	95% CI	*p*-value	HR	95% CI	*p*-value
PD	0.98	0.80–1.19	0.83	0.95	0.77–1.18	0.66
Age	n/a	n/a	n/a	0.99	0.98–1.00	**<0.01**
Female gender	n/a	n/a	n/a	0.87	0.73–1.05	0.14
Dutch nationality	n/a	n/a	n/a	1.00	0.90–1.11	0.95
Employment history	n/a	n/a	n/a	1.85	1.55–2.21	**<0.01**
Cumulative number of hours paid for competitive employment, median (IQR) worker sample (*n* = 534)	686.5 (211.0–1404.0)			781.5 (261.0–1640.5)		
Cumulative number of hours paid for competitive employment, mean (SD) worker sample (*n* = 534)	945.0 (915.5)			1089.8 (1033.3)		
	Model 1^c^			Model 2^c^		
	IRR	95% CI	*p*-value	IRR	95% CI	*p*-value
PD	0.86	0.71–1.10	0.15	0.85	0.69–1.05	0.13
Age	n/a	n/a	n/a	1.00	0.99–1.01	0.92
Female gender	n/a	n/a	n/a	0.94	0.78–1.13	0.51
Dutch nationality	n/a	n/a	n/a	0.88	0.79–0.98	**0.02**
Employment history	n/a	n/a	n/a	1.19	0.99–1.43	0.07
Cumulative number of hours paid for competitive employment, median (IQR) total sample (*n* = 1408)	0,0 (0.0–359.0)			0,0 (0.0–458.0)		
Cumulative number of hours paid for competitive employment, mean (SD) total sample (*n* = 1408)	335.4 (723.8)			414.4 (828.0)		
	Model 1 ^c^			Model 2 ^c^		
	IRR	95% CI	*p-*value	IRR	95% CI	*p-*value
PD	0.81	0.72–0.91	**<0.01**	0.84	0.73–0.95	**<0.01**
Age	n/a	n/a	n/a	1.00	0.98–0.99	**<0.01**
Female gender	n/a	n/a	n/a	0.99	0.88–1.11	0.87
Dutch nationality	n/a	n/a	n/a	0.92	0.86–0.99	**0.03**
Employment history	n/a	n/a	n/a	1.48	1.32–1.66	**<0.01**

*PD* Personality disorder, *Other SMI* Other severe mental illness, *IPS* Individual Placement and Support. Other SMI is reference

*OR* Odds ratio, 95%, *HR* Hazard ratio, *IRR* Incidence Rate Ratio of negative binomial regression, *CI* 95% confidence interval

n/a: not applicable

Significant p-values highlighted in bold

Model 1: unadjusted model

Model 2: adjusted for age, gender, nationality and employment history

^a^ Logistic regression

^b^ Cox regression

^c^ Negative binomial regression

### Time to gaining employment

**Fig. 2:**
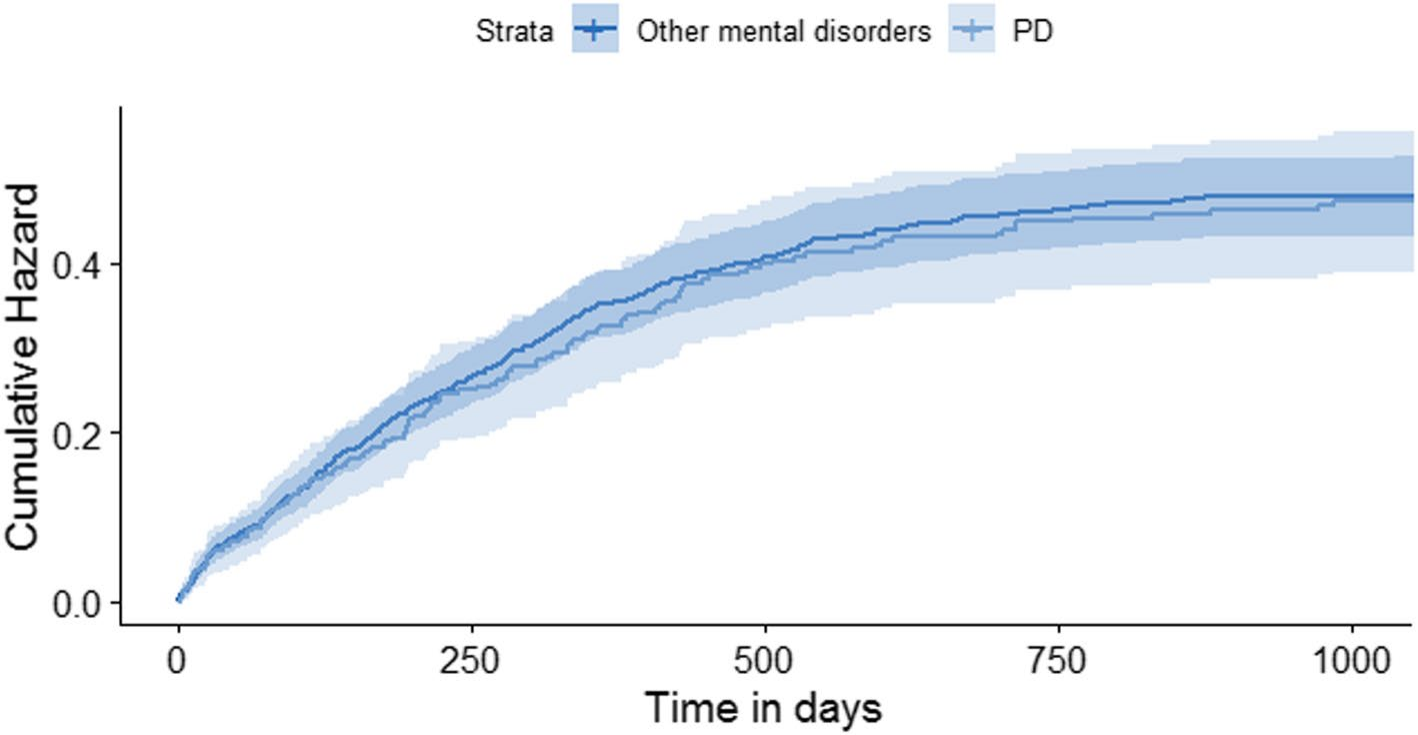
Cumulative hazard of time in days to first job in IPS (*n* = 1408). IPS: Individual Placement and Support; PD: Personality Disorder; other SMI: other severe mental illness

The data describing time to gaining employment in days of the total sample was skewed to the right. Table 3 shows that the median time to gaining employment for those gaining employment (37.9%) was 195.5 days (mean number of days 252.5) in the PD group and 178.5 days (mean number of days 234.6) in the other mental disorders group (HR = 0.98, 95% CI 0.80 to 1.19, *p* = 0.83) (Fig. 2). Again in survival analysis, although age (HR = 0.99, 95% CI 0.98 to 1.00, *p* = <0.01) and employment history (HR = 1.85, 95% CI 1.55 to 2.21, *p* = <0.01) were significantly associated with time to gaining employment in IPS, adjustment for age, gender, nationality and employment history did not affect the hazard ratio for group (HR = 0.95, 95% CI 0.77 to 1.18, *p* = 0.66) (Table 3).

### Cumulative number of hours employed

The median number of hours employed for those in competitive employment (*n* = 534) was 686.5 h in the PD group and 781.5 h in the other mental disorders group. Although nationality was associated with number of hours in competitive employment in IPS (IRR = 0.88, 95% CI 0.79 to 0.98, *p* = 0.02), adjustment for age, gender, nationality and employment history did not affect the results for group (IRR = 0.85, 95% CI 0.69 to 1.05, *p* = 0.13) (Table 3). However, when analysing the median number of hours employed for those in competitive employment within the total sample (*n* = 1408), participants with PDs had worked significantly less hours compared to those with other mental disorders (IRR = 0.81, 95% CI 0.72 to 0.91, *p* = <0.01). Also, when adjusting for age, gender, nationality and employment history (IRR = 0.84, 95% CI 0.73 to 0.95, *p* = <0.01).

Most sensitivity analyses showed similar patterns in the same direction with non-significant group differences (Additional files 1, 2 and 3). Only the sensitivity analysis that excluded the cases with ongoing IPS trajectories (*n* = 274) showed a difference between groups in cumulative number of hours employed (Additional file 1). Participants with other mental disorders had worked significantly more hours compared to those with PDs (IRR = 0.64, 95% CI 0.48 to 0.85, *p* = <0.01), also when adjusting for age, gender, nationality and employment history (IRR = 0.63, 0.46 to 0.86, *p* = <0.01). Additionally, we analysed all work outcomes for the group with a primary (*n* = 266, 79,4% of PD group) and secondary PD diagnosis (*n* = 69, 20,6% of PD group). This did not result in any differences in outcomes on gaining and time to employment nor in the cumulative number of hours worked between the groups (Additional file 4).

## Discussion

This study tested in a large, well-documented patient sample whether the relative impact of IPS differs between participants with PDs and participants with other mental disorders based on diagnosis. Patient groups did not differ in (time to) gaining employment nor in maintaining it, suggesting that IPS is no less effective a method of supported employment in PDs than in other mental disorders.

Particular strengths of the present study are the large sample size and our ability to link unique datasets on the nationwide implementation of IPS in the Netherlands over time. However, there are also limitations to consider. First, this was a prospective observational study, which implies that findings are open to bias and causal inference is limited. Second, due to differences in inclusion period for both data registers, misclassification may have occurred for some participants (see Methods). However, because PDs are longstanding disorders [24] that often remain underreported [25], the current effect sizes may be actually underestimated. As overdiagnosis cannot be ruled out, we have performed a sensitivity analysis with exclusion of participants of whom the last registered diagnosis was not a PD although they were assigned to the PD group. This did not result in any difference in outcomes on employment. Third, in line with a previous publication on the data [31] and the regulations of UWV we used a threshold of obtaining employment for 1 h or more. Because maintaining employment is a key concern in patients with PDs we have additionally studied time in employment in cumulative number of hours worked between groups to compensate. Fourth, comorbidity with other disorders contributes to occupational dysfunction in PDs [7, 32], yet we had insufficient data to study these effects in the present study. Fifth, educational status is a confounder and should be tested in IPS studies. Unfortunately, this information was missing from the data and could therefore not be included in the analyses. Sixth, the quality of IPS is determined by IPS fidelity and therefore an important measure of quality control. Unfortunately, data on IPS fidelity was not available in the present cohort. Seventh, although we could work with a large cohort, this cohort was based on an administrative dataset. The disadvantage of using administrative datasets is that we could not control data collection nor metrics. Finally, to study the actual effect of IPS in PDs (compared to other mental disorders), a control condition (e.g. treatment as usual) would be needed for both groups.

Using the largest and best-documented registry-based cohort available to date, we confirm and extend the findings of our previous exploratory study, by showing that not only (time in) gaining employment is equal between PDs and other mental disorders, but also the total number of hours worked [33]. Although ample studies show effectiveness of IPS compared to treatment as usual, review studies suggest that augmentations to a standard IPS program that improve cognitive and psychosocial skills could improve occupational outcomes even more [34–37]. This may be especially the case for patients with PDs in whom an additional social skills training may improve interpersonal functioning and problem solving at work.

## Conclusions

This study makes an important contribution to both mental health care and occupational health by showing that IPS may be effective as a means of supported employment in patients with PDs. PDs are common, debut in adolescence or early adulthood and persist over longer periods of time. Occupational functioning is thought to be crucial to the recovery of patients with PDs [38], and the immense societal costs associated with PDs are largely driven by loss of economic productivity [39, 40]. A next step would be to improve the availability and the use of IPS among patients with PDs. Furthermore, IPS should be tested within the treatment regimen of specific PD treatments and together with PD-specific augmentations to the standard IPS trajectory.

## Supplementary Information

**Additional file 1.** Employment outcomes of IPS participants and associations of employment with group without ongoing IPS trajectories (*n* = 1148).

**Additional file 2.** Employment outcomes of IPS participants and associations of employment with group, without participants of which the last registered diagnosis was not PD (*n* = 1312).

**Additional file 3.** Employment outcomes of IPS participants and associations of employment with group and double IPS trajectories (*n* = 1433).

**Additional file 4.** Employment outcomes of IPS participants and associations of employment with primary or secondary PD diagnosis group (*n* = 335).

**Additional file 5.** Personality disorder clusters within personality disorders group (*n* = 335).

## Data Availability

This dataset consists of two linked datasets that were made available for study purposes by the Dutch Employee Insurance Agency and Statistics Netherlands. These datasets are not publicly available. Under certain conditions, these microdata are accessible for statistical and scientific research. For further information: microdata@cbs.nl.

## Abbreviations

IPS: Individual Placement and Support
PD: Personality disorder
CI: Confidence interval
HR: Hazard ratio
IRR: Incidence rate ratio
BPD: Borderline personality disorder
UWV: Employee Insurance Agency
CBS: Statistics Netherlands
DSM: Diagnostic and Statistical Manual of Mental Disorders
SMI: Severe mental illness
OR: Odds ratio
