# Supplementary material for: Individual placement and support and employment in personality disorders: a registry based cohort study
Source: BMC Psychiatry. 2022 Mar 17;22:188. doi: 10.1186/s12888-022-03823-4 (PMC8932290; doi:10.1186/s12888-022-03823-4)
Supplement: Supplementary file 1 — Additional file 1. Employment outcomes of IPS participants and associations of employment with group without ongoing IPS trajectories (n = 1148). [file 12888_2022_3823_MOESM1_ESM.docx]

Additional file 1. Employment outcomes of IPS participants and associations of employment with group without ongoing IPS trajectories (n=1,148).

|  | PD | | | Other SMI | | |
| --- | --- | --- | --- | --- | --- | --- |
| Finding competitive employment, n (%) | 60 (22.3) | | | 214 (24.3) | | |
|  | Model 1ᵃ | | | Model 2ᵃ | | |
|  | OR | 95% CI | p-value | OR | 95% CI | p-value |
| PD | 0.89 | 0.64-1.24 | 0.49 | 0.94 | 0.66-1.32 | 0.71 |
| Age | n/a | n/a | n/a | 0.98 | 0.96-0.99 | **<0.01** |
| Female gender | n/a | n/a | n/a | 0.89 | 0.66-1.20 | 0.46 |
| Dutch nationality | n/a | n/a | n/a | 1.12 | 0.94-1.32 | 0.21 |
| Employment history | n/a | n/a | n/a | 1.91 | 1.45-2.54 | **<0.01** |
| Time to gaining competitive employment in days, median (IQR) worker sample (n=274) | 199,0 (56.5 – 378.0) | | | 174,5 (73.0 – 331.0) | | |
| Time to gaining competitive employment in days, mean (SD) worker sample (n=274) | 260.3 (242.9) | | | 232.9 (205.2) | | |
| Time to gaining employment in days, total sample | Model 1ᵇ | | | Model 2ᵇ | | |
|  | HR | 95% CI | p-value | HR | 95% CI | p-value |
| PD | 0.90 | 0.68-1.20 | 0.48 | 0.93 | 0.69-1.25 | 0.62 |
| Age | n/a | n/a | n/a | 0.98 | 0.97-0.99 | **<0.01** |
| Female gender | n/a | n/a | n/a | 0.88 | 0.68-1.14 | 0.33 |
| Dutch nationality | n/a | n/a | n/a | 1.09 | 0.94-1.25 | 0.26 |
| Employment history | n/a | n/a | n/a | 1.77 | 1.38-2.26 | **<0.01** |
| Cumulative number of hours paid for competitive employment, median (IQR) worker (n=274) | 341,5 (88.5 – 767.5) | | | 397,0 (104.0 – 1083.0) | | |
| Cumulative number of hours paid for competitive employment, mean (SD) worker (n=274) | 492.1 (538.3) | | | 774.5 (927.7) | | |
|  | Model 1ᶜ | | | Model 2ᶜ | | |
|  | IRR | 95% CI | p-value | IRR | 95% CI | p-value |
| PD | 0.64 | 0.48-0.85 | **<0.01** | 0.63 | 0.46-0.86 | **<0.01** |
| Age | n/a | n/a | n/a | 1.00 | 0.99-1.02 | 0.98 |
| Female gender | n/a | n/a | n/a | 0.92 | 0.71-1.20 | 0.54 |
| Dutch nationality | n/a | n/a | n/a | 0.93 | 0.80-1.08 | 0.32 |
| Employment history | n/a | n/a | n/a | 1.23 | 0.93-1.62 | 0.14 |

PD: Personality disorder; Other SMI: Other Severe mental illness; IPS: Individual Placement and Support. Other SMI is reference

OR: Odds ratio; 95%, HR: Hazard ratio, IRR: Incidence Rate Ratio of negative binomial regression, CI: 95% confidence interval.

n/a: not applicable.

Model 1: unadjusted model

Model 2: adjusted for age, gender, nationality and employment history;

ᵃ Logistic regression;

ᵇ Cox regression;

ᶜ Negative binomial regression.
