# Supplementary material for: Individual placement and support and employment in personality disorders: a registry based cohort study
Source: BMC Psychiatry. 2022 Mar 17;22:188. doi: 10.1186/s12888-022-03823-4 (PMC8932290; doi:10.1186/s12888-022-03823-4)
Supplement: Supplementary file 2 — Additional file 2. Employment outcomes of IPS participants and associations of employment with group, without participants of which the last registered diagnosis was not PD (n = 1312). [file 12888_2022_3823_MOESM2_ESM.docx]

Additional file 2. Employment outcomes of IPS participants and associations of employment with group, without participants of which the last registered diagnosis was not PD (n=1,312).

|  | PD | | | Other SMI | | |
| --- | --- | --- | --- | --- | --- | --- |
| Finding competitive employment, n (%) | 87 (36.4) | | | 408 (38.0) | | |
|  | Model 1ᵃ | | | Model 2ᵃ | | |
|  | OR | 95% CI | p-value | OR | 95% CI | p-value |
| PD | 0.93 | 0.70-1.25 | 0.64 | 0.92 | 0.67-1.25 | 0.59 |
| Age | n/a | n/a | n/a | 0.98 | 0.97-0.99 | **<0.01** |
| Female gender | n/a | n/a | n/a | 0.83 | 0.65-1.07 | 0.15 |
| Dutch nationality | n/a | n/a | n/a | 0.99 | 0.86-1.14 | 0.87 |
| Employment history | n/a | n/a | n/a | 2.15 | 1.71-2.71 | **<0.01** |
| Time to gaining competitive employment in days, median (IQR) worker sample (n=495) | 197,0 (62.0 – 351.5) | | | 178,5 (76.0 – 341.0) | | |
| Time to gaining competitive employment in days, mean (SD) worker sample (n=495) | 234.7 (207.1) | | | 234.6 (204.1) | | |
| Time to gaining employment in days, total sample | Model 1ᵇ | | | Model 2ᵇ | | |
|  | HR | 95% CI | p-value | HR | 95% CI | p-value |
| PD | 0.95 | 0.75-1.19 | 0.64 | 0.93 | 0.73-1.18 | 0.53 |
| Age | n/a | n/a | n/a | 0.99 | 0.98-1.00 | **<0.01** |
| Female gender | n/a | n/a | n/a | 0.84 | 0.70-1.02 | 0.08 |
| Dutch nationality | n/a | n/a | n/a | 0.99 | 0.88-1.10 | 0.80 |
| Employment history | n/a | n/a | n/a | 1.86 | 1.55-2.24 | **<0.01** |
| Cumulative number of hours paid for competitive employment, median (IQR) worker sample (n=495) | 707,0 (156.0 – 1373.0) | | | 781,5 (261.0 – 1640.5) | | |
| Cumulative number of hours paid for competitive employment, mean (SD) worker sample (n=495) | 905.4 (863.4) | | | 1089.8 (1033.3) | | |
|  | Model 1ᶜ | | | Model 2ᶜ | | |
|  | IRR | 95% CI | p-value | IRR | 95% CI | p-value |
| PD | 0.83 | 0.66-1.05 | 0.12 | 1.23 | 0.96-1.58 | 0.10 |
| Age | n/a | n/a | n/a | 1.00 | 0.99-1.01 | 0.62 |
| Female gender | n/a | n/a | n/a | 1.00 | 0.82-1.22 | 1.00 |
| Dutch nationality | n/a | n/a | n/a | 0.88 | 0.79-0.99 | **0.03** |
| Employment history | n/a | n/a | n/a | 1.19 | 0.98-1.44 | 0.08 |

PD: Personality disorder; Other SMI: Other Severe mental illness; IPS: Individual Placement and Support. Other SMI is reference

OR: Odds ratio; 95%, HR: Hazard ratio, IRR: Incidence Rate Ratio of negative binomial regression, CI: 95% confidence interval.

n/a: not applicable.

Model 1: unadjusted model

Model 2: adjusted for age, gender, nationality and employment history;

ᵃ Logistic regression;

ᵇ Cox regression;

ᶜ Negative binomial regression.
