# Supplementary material for: Individual placement and support and employment in personality disorders: a registry based cohort study
Source: BMC Psychiatry. 2022 Mar 17;22:188. doi: 10.1186/s12888-022-03823-4 (PMC8932290; doi:10.1186/s12888-022-03823-4)
Supplement: Supplementary file 3 — Additional file 3. Employment outcomes of IPS participants and associations of employment with group and double IPS trajectories (n = 1433). [file 12888_2022_3823_MOESM3_ESM.docx]

Additional file 3. Employment outcomes of IPS participants and associations of employment with group and double IPS trajectories (n=1,433).

|  | PD | | | Other SMI | | |
| --- | --- | --- | --- | --- | --- | --- |
| Finding competitive employment, n (%) | 126 (37.5) | | | 413 (37.6) | | |
|  | Model 1ᵃ | | | Model 2ᵃ | | |
|  | OR | 95% CI | p-value | OR | 95% CI | p-value |
| PD | 0.99 | 0.77-1.28 | 0.96 | 0.98 | 0.75-1.28 | 0.86 |
| Age | n/a | n/a | n/a | 0.98 | 0.97-0.99 | **<0.01** |
| Female gender | n/a | n/a | n/a | 0.88 | 0.69-1.11 | 0.27 |
| Dutch nationality | n/a | n/a | n/a | 1.01 | 0.88-1.16 | 0.94 |
| Employment history | n/a | n/a | n/a | 2.19 | 1.75-2.73 | **<0.01** |
| Time to gaining competitive employment in days worker sample, median (IQR) (n=539) | 195,5 (75.0 – 378.0) | | | 179,0 (76.0 – 341.0) | | |
| Time to gaining competitive employment in days, mean (SD) worker sample (n=539) | 252.5 (223.7) | | | 234.1 (203.5) | | |
| Time to gaining employment in days, total sample | Model 1ᵇ | | | Model 2ᵇ | | |
|  | HR | 95% CI | p-value | HR | 95% CI | p-value |
| PD | 0.99 | 0.81-1.20 | 0.89 | 0.96 | 0.78-1.18 | 0.68 |
| Age | n/a | n/a | n/a | 0.99 | 0.98-1.00 | **<0.01** |
| Female gender | n/a | n/a | n/a | 0.87 | 0.73-1.05 | 0.14 |
| Dutch nationality | n/a | n/a | n/a | 1.00 | 0.90-1.11 | 0.96 |
| Employment history | n/a | n/a | n/a | 1.88 | 1.57-2.24 | **<0.01** |
| Cumulative number of hours paid for competitive employment, median (IQR) worker sample (n=539) | 686,5 (211.0 – 1404.0) | | | 782,0 (261.0 – 1648.0) | | |
| Cumulative number of hours paid for competitive employment, mean (SD) worker sample (n=539) | 945.0 (915.5) | | | 1093.3 (1035.9) | | |
|  | Model 1ᶜ | | | Model 2ᶜ | | |
|  | IRR | 95% CI | p-value | IRR | 95% CI | p-value |
| PD | 0.86 | 0.71-1.05 | 0.15 | 0.85 | 0.69-1.05 | 0.12 |
| Age | n/a | n/a | n/a | 1.00 | 0.99-1.01 | 0.99 |
| Female gender | n/a | n/a | n/a | 0.94 | 0.78-1.13 | 0.50 |
| Dutch nationality | n/a | n/a | n/a | 0.87 | 0.78-0.97 | **0.02** |
| Employment history | n/a | n/a | n/a | 1.19 | 0.98-1.43 | 0.07 |

PD: Personality disorder; Other SMI: Other Severe mental illness; IPS: Individual Placement and Support. Other SMI is reference

OR: Odds ratio; 95%, HR: Hazard ratio, IRR: Incidence Rate Ratio of negative binomial regression, CI: 95% confidence interval.

n/a: not applicable.

Model 1: unadjusted model

Model 2: adjusted for age, gender, nationality and employment history;

ᵃ Logistic regression;

ᵇ Cox regression;

ᶜ Negative binomial regression.
