# Supplementary material for: Individual placement and support and employment in personality disorders: a registry based cohort study
Source: BMC Psychiatry. 2022 Mar 17;22:188. doi: 10.1186/s12888-022-03823-4 (PMC8932290; doi:10.1186/s12888-022-03823-4)
Supplement: Supplementary file 4 — Additional file 4. Employment outcomes of IPS participants and associations of employment with primary or secondary PD diagnosis group (n = 335). [file 12888_2022_3823_MOESM4_ESM.docx]

Additional file 4. Employment outcomes of IPS participants and associations of employment with primary or secondary PD diagnosis group (n=335).

|  | Primary PD diagnosis | | | Secondary PD diagnosis | | |
| --- | --- | --- | --- | --- | --- | --- |
| Finding competitive employment, n (%) | 104 (39.1) | | | 22 (31.9) | | |
|  | Model 1ᵃ | | | Model 2ᵃ | | |
|  | OR | 95% CI | p-value | OR | 95% CI | p-value |
| PD | 0.73 | 0.42-1.28 | 0.27 | 0.76 | 0.43-1.37 | 0.36 |
| Age | n/a | n/a | n/a | 0.98 | 0.95-1.00 | 0.09 |
| Female gender | n/a | n/a | n/a | 0.90 | 0.56-1.43 | 0.65 |
| Dutch nationality | n/a | n/a | n/a | 1.23 | 0.85-1.77 | 0.27 |
| Employment history | n/a | n/a | n/a | 2.00 | 1.26-3.16 | **<0.01** |
| Time to gaining competitive employment in days, median (IQR) worker sample (n=126) | 170,0 (103.0 – 452.0) | | | 196,5 (73.0 – 357.5) | | |
| Time to gaining competitive employment in days, mean (SD) worker sample (n=126) | 310.5 (295.0) | | | 240.2 (205.2) | | |
| Time to gaining employment in days, total sample | Model 1ᵇ | | | Model 2ᵇ | | |
|  | HR | 95% CI | p-value | HR | 95% CI | p-value |
| PD | 0.76 | 0.48-1.21 | 0.25 | 0.80 | 0.50-1.28 | 0.35 |
| Age | n/a | n/a | n/a | 0.98 | 0.96-1.00 | 0.10 |
| Female gender | n/a | n/a | n/a | 0.87 | 0.60-1.25 | 0.45 |
| Dutch nationality | n/a | n/a | n/a | 1.16 | 0.88-1.51 | 0.29 |
| Employment history | n/a | n/a | n/a | 1.75 | 1.21-2.52 | **<0.01** |
| Cumulative number of hours paid for competitive employment, median (IQR) worker sample (n=126) | 682,5 (213.5 – 1462.5) | | | 705,5 (133.0 – 1320.0) | | |
| Cumulative number of hours paid for competitive employment, mean (SD) worker sample (n=126) | 955.4 (930.9) | | | 895.9 (857.7) | | |
|  | Model 1ᶜ | | | Model 2ᶜ | | |
|  | IRR | 95% CI | p-value | IRR | 95% CI | p-value |
| PD | 0.94 | 0.59-1.49 | 0.79 | 0.85 | 0.52-1.39 | 0.51 |
| Age | n/a | n/a | n/a | 1.01 | 0.99-1.03 | 0.45 |
| Female gender | n/a | n/a | n/a | 0.95 | 0.64-1.39 | 0.77 |
| Dutch nationality | n/a | n/a | n/a | 0.83 | 0.64-1.08 | 0.17 |
| Employment history | n/a | n/a | n/a | 1.31 | 0.88-1.96 | 0.19 |

PD: Personality disorder; IPS: Individual Placement and Support. Secondary PD diagnosis is reference

OR: Odds ratio; 95%, HR: Hazard ratio, IRR: Incidence Rate Ratio of negative binomial regression, CI: 95% confidence interval.

n/a: not applicable.

Model 1: unadjusted model

Model 2: adjusted for age, gender, nationality and employment history;

ᵃ Logistic regression;

ᵇ Cox regression;

ᶜ Negative binomial regression.
