# Supplementary material for: Individual placement and support and employment in personality disorders: a registry based cohort study
Source: BMC Psychiatry. 2022 Mar 17;22:188. doi: 10.1186/s12888-022-03823-4 (PMC8932290; doi:10.1186/s12888-022-03823-4)
Supplement: Supplementary file 5 — Additional file 5. Personality disorder clusters within personality disorders group (n = 335). [file 12888_2022_3823_MOESM5_ESM.docx]

Additional file 5. Personality disorder clusters within personality disorders group (n=335).

| Personality disorder | Total within personality disorder group n (%) |
| --- | --- |
| Cluster A | 17 (5.1) |
| Cluster B | 106 (31.6) |
| Cluster C | 79 (23.6) |
| Personality disorder not otherwise specified | 133 (39.7) |
